# Antioxidant Polyphenols from *Lespedeza bicolor* Turcz. Honey: Anti-Inflammatory Effects on Lipopolysaccharide-Treated RAW 264.7 Macrophages

**DOI:** 10.3390/antiox12101809

**Published:** 2023-09-28

**Authors:** Caijun Ren, Qiangqiang Li, Teng Luo, Mirko Betti, Miao Wang, Suzhen Qi, Liming Wu, Liuwei Zhao

**Affiliations:** 1 State Key Laboratory of Resource Insects, Institute of Apicultural Research, Chinese Academy of Agricultural Sciences, Beijing 100093, China; 82101221123@caas.cn (C.R.); liqiangqiang@caas.cn (Q.L.); wangmiao03@caas.cn (M.W.); qisuzhen@caas.cn (S.Q.); 2 Institute of NBC Defence, Beijing 102205, China; yunyuebaqian@163.com; 3 Department of Agricultural Food and Nutritional Science, Faculty of Agricultural, Life & Environmental Sciences, University of Alberta, Edmonton, AB T6G 2P5, Canada; mirko.betti@ualberta.ca; 4 Risk Assessment Laboratory for Bee Products Quality and Safety of Ministry of Agriculture, Beijing 100093, China

**Keywords:** *Lespedeza bicolor* Turcz. honey, polyphenols, antioxidative activity, anti-inflammatory activity

## Abstract

Although the honey produced by *Lespedeza bicolor* Turcz. is precious because of its medicinal value, its pharmacological mechanism is still unclear. Here, its anti-inflammatory and antioxidant functions on lipopolysaccharide (LPS)-treated murine RAW 264.7 macrophages were analyzed using targeted and non-targeted metabolomics. Results showed that twelve polyphenols were identified in *L. bicolor* honey using UHPLC-QQQ-MS/MS. *L. bicolor* honey extract could scavenge the free radicals DPPH^•^ and ABTS^+^ and reduce Fe^3+^. Furthermore, pretreatment with *L. bicolor* honey extract significantly decreased NO production; suppressed the expression of *COX-2*, *IL-10*, *TNF-α*, and *iNOS*; and upregulated *HO-1′*s expression in the cells with LPS application. UHPLC-Q-TOF-MS/MS-based metabolomics results revealed that *L. bicolor* honey extract could protect against inflammatory damage caused by LPS through the reduced activation of sphingolipid metabolism and necroptosis pathways. These findings demonstrate that *L. bicolor* honey possesses excellent antioxidant and anti-inflammatory activities.

## 1. Introduction

Cells can produce free radicals during normal metabolism; however, once normal metabolism is disrupted by stress, excessive free radicals can be produced, leading to cell damage [1,2]. Oxidative stress and the excess of free radicals can be caused by several factors, one of which is the inflammation that occurs during the body’s natural immune response [3]. The progression of asthma, cancer, chronic fatigue syndrome, and infertility was reported to be accelerated by inflammatory and oxidative stress [4]. When the body suffers harm from factors such as physical injury, toxins, and microorganisms [5,6], macrophage immune cells come into prominent play not only during the inflammatory response but also in the repair of tissue damage [7]. Hence, the inflammatory response is considered as a beneficial process to help the body defend against pathogens; however, excessive inflammation can induce fatal tissue damage [8].

Macrophage cells are widely distributed in the body and play the role of phagocyting foreign bodies, clearing bacteria, and fighting inflammatory diseases. Once inflammatory and oxidative stress progress happen, macrophage cells are activated along with the ossification of the cellular membrane. Then, macrophage cells begin to secrete proinflammatory cytokines [7]. The RAW 264.7 cell line is extensively used as a reliable cell model to study the physiological function of macrophages. Lipopolysaccharide (LPS) as an effective stimulator in immune defense is applied to the murine macrophage cell line RAW 264.7 to study its anti-inflammatory or antioxidant functions [9]. LPS stimulated RAW 264.7 cells to secrete proinflammatory cytokines such as *TNF-α*, *iNOS*, and interleukin-*1β* (*IL-1β*), leading to the NO generation. The latter can result in excessive nitric oxide (NO) [10]. While NO plays an important role in intracellular signal transduction [11], as a free radical, its excess accumulation during the inflammatory response can lead to severe toxicity [1]. Currently, anti-inflammatory drugs are used to regulate the levels of inflammatory cytokines to reduce inflammation but are associated with some side effects [12]. Identification of functional foods and dietary supplements that can reduce inflammation is an active research area.

During the early stage of the inflammatory response, inflammatory mediator *TNF-α* can be accumulated. This activates the production of neutrophil granulocytes and leukomonocytes, enhancing the permeability of vascular endothelium and accelerating the secretion of *IL-10* and *COX-2* [13]. *IL-10*, which is produced as a feedback factor from the stress response under *TNF-α* or other toxins stimulating macrophages, can inhibit the inflammatory process such as the migration of inflammatory cytokines [14]. As a key factor in promoting inflammation, the activation of *COX-2* led to aggravated inflammatory reactions and damaged tissues [15].

LPS can induce the excessive expression of *iNOS* transcripts to promote NO production and cause dystrophy or hypoxia in macrophages [16]. While *iNOS* can make NO as a free radical during oxidative stress, it helps the macrophages defend against pathogens under normal conditions [17]. Another important marker is *Heme oxygenase-1* (*HO-1*), which encodes an important antioxidative enzyme that shows increased expression during oxidative stress. *HO-1* effectively scavenges ROS, including peroxides, peroxynitrite, hydroxyl radicals, and superoxide radicals [18]. *HO-1* expression is activated by LPS, which helps inhibit inflammation and oxidative damage by reducing NO production and protecting macrophages against oxidative stress [19]. Therefore, the mRNA levels of these cytokine genes may work as valid indicators to evaluate antioxidant and anti-inflammatory effects.

*Lespedeza bicolor* Turcz. (*L. bicolor*) blooms have been traditionally used in medicine as a means of removing toxins, replenishing energy, and regulating symptoms associated with diabetes. It has also been used to treat acute and chronic nephritis, azotemia, and promote diuresis [20]. More recently, its antioxidant activity has been studied and has shown promise as a treatment for endothelial dysfunction [21,22]. Honeys produced by honeybees (*Apis mellifera*) from medicinal plants such as clover (*Trifolium alexandrinum* L.) and Manuka (*Leptospermum scoparium*) have antioxidant and anti-inflammatory activities [23,24]. In particular, Manuka honey was shown to modulate the production of nuclear factor caspase 3, p-p38, and p-Erk 1/2 proteins to protect macrophages against LPS-induced inflammation [25]. Manuka plants exert antibacterial, anti-inflammatory, and antioxidant activities, contributing to the bioactivities of manuka honey [26]. The flowering Asian shrub *L. bicolor* plant also has many biological activities. We consider that *L. bicolor* honey has potential antioxidant and anti-inflammatory activities for exploitation.

Therefore, in this study, our goal is to use an LPS-induced RAW 264.7 cell model to identify the antioxidant and anti-inflammatory functions of *L. bicolor* honey and its underlying molecular mechanisms. To this end, we first investigated the composition and levels of major polyphenols in *L. bicolor* honey using UHPLC-Q-TOF-MS/MS and UHPLC-QQQ-MS/MS. We then detected free radical (including DPPH^•^, ABTS^+^, and Fe^3+^) scavenging abilities and determined the expression levels of a number of oxidation- and inflammation-related genes to assess the antioxidant and anti-inflammatory functions that occur in the *L. bicolor* honey. Finally, cellular metabolites were analyzed based on the UHPLC-Q-TOF-MS/MS results.

## 2. Materials and Methods

### 2.1. Chemical Reagents

Chemical reagents like ethanol, methanol, acetonitrile, and formic acid were all of chromatographic grade from Thermo Fisher Scientific Inc. (Waltham, MA, USA). Using a Milli-Q Plus instrument from Merk KGaA, 18.2 MΩ.cm water was made (Darmstadt, Germany1). Folin–Ciocalteu and aluminum nitrate were supplied by Solarbio Science & Technology Co., Ltd. (Beijing, China). Chemical standards including chlorogenic acid, ferulic acid, vitexin, rutin, gallic acid, myricitrin, morin, glycitein, wogonin, kaempferol-3-O-sophoroside, liquiritigenin, and butin were obtained from Yuanye Biological Technology Co., Ltd. (Shanghai, China), with >98% purity. The extract was concentrated on Bond Elut PPL 6 cc, 500 mg C18 solid phase extraction (SPE) cartridge that was purchased from Agilent Technology Co., Ltd. (Beijing, China). High-glucose (4.5 g/L) Dulbecco’s modified eagle’s medium (DMEM), which contains 10% (*v*/*v*) heat-inactivated fetal bovine serum, 100 µg/mL of streptomycin, and 100 U/mL of penicillin, was obtained from Gibco Laboratories (Carlsbad, CA, USA). *Escherichia coli* 0111: B4 LPS was a Sigma product (St. Louis, MO, USA).

### 2.2. Collection and Processing of Honey

Honey samples were collected from an apiary located in an *L. bicolor* plantation (Da Hinggan Ling Prefecture, China) in August 2020. Palynological analysis showed that *L. bicolor* honey contained ~70% *L. bicolor* pollen grains. It meant *L. bicolor* honey was monofloral honey that met the pollen grain threshold (≥45%) [25].

A total of 5 g of honey was dissolved in 10 mL of water, and after a one-minute vortex, the solution was centrifuged for five minutes at 8000× g, and the supernatant was collected for the following use. The SPE C_18_ cartridges were used to enrich the polyphenol compounds in the supernatant, and then, methanol was used as an elution agent to collect polyphenol compounds from the cartridges [25]. Finally, the eluted solution was dried with nitrogen gas and subsequently dissolved with 1 mL of ethanol to obtain a final solution (200 mg/mL) for the following study.

### 2.3. Determination of Polyphenol and Flavonoid Content

In our previous study, the total content of polyphenol in the *L. bicolor* honey extract was examined using the Folin–Ciocalteu method, and it was defined as gallic acid equivalents (GAEs) [27]. Additionally, the total content of flavonoids from honey was determined using the aluminum nitrate method, and it was taken as quercetin equivalents (QEs) [27].

### 2.4. UHPLC-Q-TOF-MS/MS Parameter

The chromatography was performed via a 1290 HPLC system, with a 2.1 × 100 mm i.d Eclipse Plus C18 column obtained from Agilent Technology Co., Ltd. (Santa Clara, CA, USA). Analytes were separated via water and acetonitrile (both A and B containing 0.1% formic acid, *v*/*v*) according to a gradient elution program (5% B for 0 min, followed by 5% B for 2 min, 100% B for 20 min, and 100% B for 25 min), with a 0.3 mL/min flow rate. Post-time was 5 min, and the column was always kept constant at 40 °C.

MS measurements were carried out via Q-TOF with an electrospray ionization source by Agilent Technology Co., Ltd. (Santa Clara, CA, USA). The conditions are as follows: gas temperature of 325 °C (at a 10 L/min flow rate), nebulizer pressure of 35 psi, sheath gas temperature of 370 °C (at a 12 L/min flow rate), fragmentor voltage of 135 V, and the acquisition ranges between *m*/*z* 100 and 1700. *m*/*z* 121.0508, *m*/*z* 922.0097, *m*/*z* 112.9855, and *m*/*z* 1033.9881 were, respectively, used for mass accuracy monitoring and calibration on positive and negative ionization modes. The RAW 264.7 cell metabolite samples were scanned in the positive ionization mode. *L. bicolor* honey samples were in the negative ionization mode. The acquired raw data files were transformed to CEF files through Mass Hunter Profinder 8.0 software from Agilent Technology Co., Ltd. (Santa Clara, CA, USA) after filtration using peak height ≥ 1500 counts and absolute height ≥ 10,000 counts. Then, the filtered data were matched to the METLIN database (a metabolite mass spectral database) of Scripps Research using Mass Profiler Professional software B.10.0 from Agilent Technology Co., Ltd., (Santa Clara, CA, USA) with thresholds of a database score > 70 and mass error < 10 ppm.

### 2.5. UHPLC-QQQ-MS/MS Analysis

UHPLC-QQQ-MS/MS was carried out via a UHPLC/MS system, with an Eclipse Plus C_18_ analytical column (2.1 mm × 100 mm, 1.8 µm), obtained from Agilent Technology Co., Ltd. (Santa Clara, CA, USA), at a 0.35 mL/min flow rate. A total of 2 µL of water + 0.1% formic acid (*v*/*v*) (A) and acetonitrile + 0.1% formic acid mobile phase (*v*/*v*) (B) were used for the mobile phase, respectively. The gradient elution process was as follows: 5% B at the beginning; followed by 5% B at the 1st min; 50% B in the 2nd min; 50% B in the 5th min; 100% B at minute 5.1; and 100% B at the 6th min. Post run time was set as 2 min. The ingredients were examined through a multiple reaction monitoring (MRM) system. The parameters for ESI source were as follows: 3.5 kV capillary voltage, 380 V fragmentor voltage, 200 °C drying gas (with a 15 L/min flow rate), 330 °C sheath gas (flow rate of 12 L/min), and 45 psi nebulizer pressure. The related compounds ionization parameters are listed in Table S1.

### 2.6. In Vitro Antioxidant Assays

The antioxidant properties of *L. bicolor* honey extract were analyzed using free radical scavenging and reducing ability assay kits to perform DPPH^•^, ABTS^+^ assay, and FRAP assay from Solarbio Biotechnology Co., Ltd. (Beijing, China).

For DPPH^•^ radical scavenging activity determination, 190 μL of 0.2 mmol/L DPPH-ethanol solution was mixed with 10 μL of a different concentration of *L. bicolor* honey extract solution, and the mixture was vortexed for 30 s, then left to stand for 30 min in the dark at room temperature. Vitamin C was used as a positive control [21]. The absorbance was detected at 517 nm.

The DPPH radical scavenging activity (%) was expressed as follows:

$$(\%)=\frac{A1-\left(A2-A0\right)}{A0}\times 100\%$$

where A_1_ is the absorbance of a mixture of 190 μL of DPPH–ethanol and 10 μL of ethanol, A_2_ is the absorbance of different concentrations of *L. bicolor* honey extract solution or positive control with DPPH–ethanol, and A_0_ is the absorbance of the control group instead of DPPH–ethanol solution.

For ABTS^+^ radical scavenging activity determination: 10 μL of different concentrations of *L. bicolor* honey extract solution was added to 190 μL of work solution (5 mL 7 mol/L ABTS solution and 88 μL 140 mmol/L K_2_S_2_O_8_ solution) and vortexed for 30 s, then the mixture was kept for 6 min in the dark. The absorbance was measured at 415 nm.

For FRAP reducing ability determination: 180 μL work solution (0.3 mol/L acetic acid buffer, 10 mmol/L TPTZ solution, and 20 mmol/L FeCl_3_ solution) were mixed with 5 μL of different concentrations of *L. bicolor* honey extract solution and vortexed for 30 s. The absorbance of the mixture was measured at 593 nm.

### 2.7. Cell Culture

#### 2.7.1. Cell Incubation and Assay

The mouse peritoneal macrophage cells, supplied by the Cell Bank of Shanghai Institute of Biochemistry and Cell Biology (Shanghai, China), were cultured in DMEM and stored with 5% CO_2_ at 37 °C at the concentration of 1 × 10^5^/mL. Then, it was pretreated with different concentrations of *L. bicolor* honey extract. Their viability was cultured in 96-well plates with 1 × 10^5^/mL of seeding density at the 4th passage and evaluated with a cell counting kit-8 from Dojindo Inc. (Kumamoto, Japan). The 450 nm absorbance was examined via microplate reader software from SpectraMax^®^ i3 Platform (Silicon, CA, USA).

#### 2.7.2. LPS-Induced NO Measurement

After growing for 24 h in 24-well plates, 1 × 10^5^/mL of seeding density RAW 264.7 cells at the 5th passage were conducted 1 h pretreatment with different concentrations of *L. bicolor* honey extract (50, 100, and 200 μg/mL) and 24 h induction with 1 µg/mL of LPS. The LPS-induced NO was detected using a NO detection kit from Beyotime Biotechnology Co., Ltd., (Shanghai, China) according to the Griess reaction. The 540 nm absorbance was checked via a microplate reader from SpectraMax^®^ i3 Platform (Silicon, CA, USA).

#### 2.7.3. RNA Extraction and qRT-PCR

After 1 h incubation with *L. bicolor* honey extract and 6 h stimulation with 1 µg/mL of LPS, the RAW 264.7 cells’ RNA were extracted using an RNA Pure Kit from Aidlab Biotechnologies Co., Ltd., (Beijing, China) and quantified via the Nano Drop 2000 system. A qRT-PCR was performed on a 7500c Real-time PCR detection system (Hangzhou, China), with specific primers (Table S2). The RT-qPCR program was set as follows: initial holding stage at 95 °C for 30 s; followed by 40 cycles at 95 °C for 5 s and 60 °C for 30 s; and then at 95 °C for 15 s, 60 °C for 60 s, and 95 °C for 1 s. The expression status of target genes was assayed using the 2^−ΔΔCt^ method, with *GAPDH* as an internal reference.

#### 2.7.4. Collection of Metabolites from LPS-Treated Cells

RAW 264.7 cells at the 5th passage were incubated in 6-well plates with 1 × 10^5^/mL of seeding density when their concentration was reached. The cells first received 1 h of pretreatment with different concentrations of *L. bicolor* honey extract. And then, they were conducted with a 24 h induction with LPS (1 µg/mL). Finally, the cells were centrifuged to precipitate cells (2500× g, 5 min), followed by a PBS wash, addition of 1 mL of methanol/acetonitrile/water mixture (2:2:1, volume ratio), and 5 min centrifugation (10,000× g, 4 °C), 1 min vortex, and the supernatant was dried with nitrogen gas after being moved into a new tube. Cellular extracts were redissolved in a 50% acetonitrile solution and ready for injection after a 0.22 μm filtering. A combined equal volume of each sample was used to obtain a quality control (QC) sample for data variance.

#### 2.7.5. UHPLC-Q-TOF-MS/MS

RAW 264.7 cell metabolites were acquired through UHPLC-Q-TOF-MS/MS, with specific parameters set according to the protocol outlined in Section 2.4.

### 2.8. Statistics

Our statistical data were presented as mean ± SD. One-way ANOVA and Tukey’s honest tests were adopted to compare the significant differences among different treatment groups in Section 3.4, Section 3.5 and Section 3.6 using IBM SPSS version 23.0 from IBM Corporation (Shanghai, China). *p* ˂ 0.05 refers to a significant difference. The screened metabolites with KEGG ID obtained from a volcano analysis were input into MetaboAnalyst 4.0 (https://www.metaboanalyst.ca, accessed on 6 June 2022), then linked to KEGG (http://www.kegg.jp, accessed on 6 June 2022) to perform metabolic pathway analyses, and *p* < 0.05 was set as a threshold. Mus musculus was chosen as the pathway organism.

## 3. Results and Discussion

### 3.1. The Polyphenols and Flavonoids Content on L. bicolor Honey

Polyphenols and flavonoids are identified as the major compounds in honey because of their medical functions, which are antioxidant, anti-inflammatory, antimicrobial, antiviral, anti-cancer, etc. [4,28]. As shown in Table S3, the total polyphenol content of *L. bicolor* honey was 147.7 ± 3.3 µg GAE/g honey, and the total flavonoid content was 8.5 ± 0.8 µg QE/g honey. Both the polyphenol and flavonoid content of *L. bicolor* honey were found to be higher as compared to other honeys. For instance, a total polyphenol content of 22.6–72.8 µg GAE/g was reported for Portuguese honey [29]; an average of 86.7 µg GAE/g was reported for Turkey rhododendron honey [30]; and 16.0–120.0 µg GAE/g for oak honey [31]. The total flavonoid content of honey from jujube, cactus, and multifloral honeys were found to be 5.7 ± 0.01, 11.6 ± 0.3, and 5.3 ± 0.2 µg QE/g, respectively [32]. The results indicate that *L. bicolor* honey contains abundant polyphenols and flavonoids, which could potentially contribute to its biological activity.

### 3.2. The Detection of Polyphenols in L. bicolor Honey

**Figure 1:**
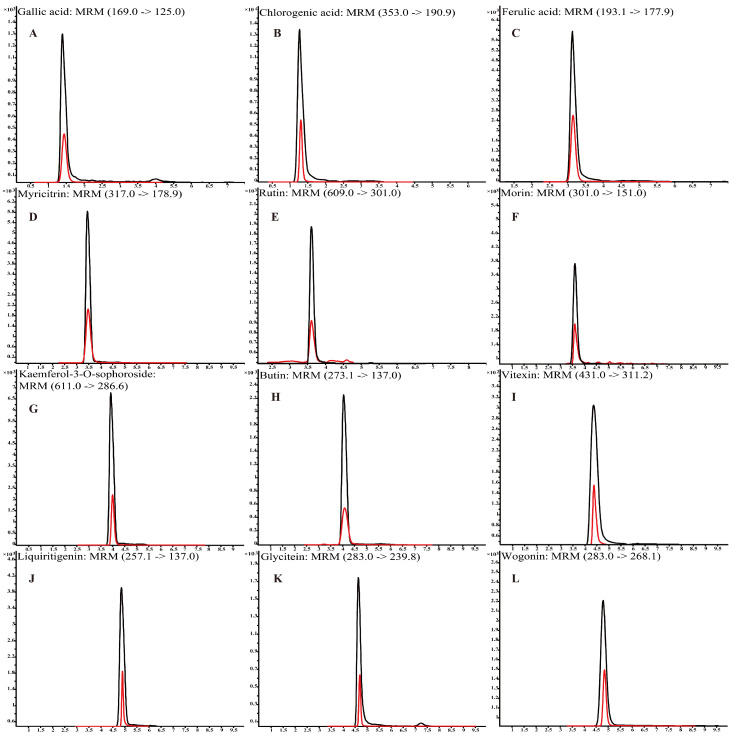
Mass spectra of 12 polyphenol compounds in *L. bicolor* honey acquired in multiple reaction monitoring (MRM) mode using UHPLC-QQQ-MS/MS. (**A**) gallic acid (169.0/125.0), (**B**) chlorogenic acid (353.0/190.9), (**C**) ferulic acid (193.1/177.9), (**D**) myricitrin (317.0/178.9), (**E**) rutin (609.0/301.0), (**F**) morin (301.0/151.0), (**G**) kaempferol-3-O-sophoroside (611.0/286.6), (**H**) butin (273.1/137.0), (**I**) vitexin (431.0/311.2), (**J**) liquiritigenin (257.1/137.0), (**K**) glycitein (283.0/139.8), and (**L**) wogonin (283.0/268.1). Black line is for standards; red line is for *L. bicolor* honey.

Polyphenols were identified using a non-targeted metabolomics analysis based on UHPLC-Q-TOF-MS/MS (Table S4). For instance, chlorogenic acid, glycitein, and vitexin were identified at a *m*/*z* of 353.0878, 283.0612, and 431.0984, respectively. The typical chromatography spectra of 12 polyphenol compounds in standard solutions and *L. bicolor* honey extract are shown in Figure 1. Further, UHPLC-QQQ-MS/MS was performed to quantify the major polyphenols in 48 *L. bicolor* honey samples from eight different apiaries. Results in Table 1 show high levels of chlorogenic acid (16.3 ± 5.7 mg/kg) and glycitein (17.3 ± 3.4 mg/kg) in *L. bicolor* honey. Chlorogenic acid belongs to a product of aerobic respiration found in the honey of nectar source plants [33]. Chlorogenic acid is a phenylpropanoid with antioxidant, antibacterial, antiviral, antihyperlipidemic, and other biological activities [34]. Glycitein is an o-methylated isoflavone with free radical scavenging activity and phytoestrogen activity that has potential application in preventing the occurrence of cerebrovascular diseases [35]. Additionally, the extract contained high levels of other flavonoids such as rutin, myricitrin, and wogonin. These results indicate that *L. bicolor* honey is rich in polyphenols.

### 3.3. In Vitro Antioxidant Activity of L. bicolor Honey

**Figure 2:**
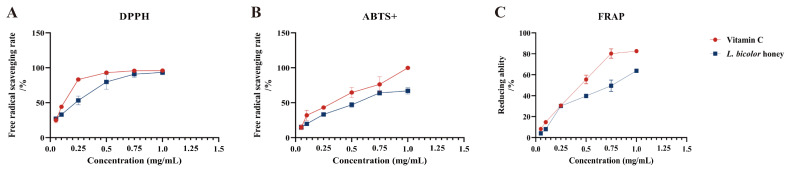
Radical scavenging and reducing abilities of *L. bicolor* honey extract. The scavenging rate of the free radicals (**A**) DPPH and (**B**) ABTS^+^ as well as (**C**) Fe^3+^ reducing ability. Vitamin C was set as positive control for excluding false positives of the experiment and referring to the antioxidant of *L. bicolor* honey extract.

Our results suggest that *L. bicolor* honey extract contains abundant phenolics such as phenolic acid (chlorogenic acid, ferulic acid, gallic acid, etc.) and flavonoids (myricitrin, rutin, vitexin, etc.), which are known to have excellent antioxidant activity. The scavenging of DPPH^•^ and ABTS^+^ and the reduction in Fe^3+^ have been commonly used to evaluate the antioxidant activities of substances [36]. Antioxidant activity was measured as a percentage of decreasing DPPH^•^, ABTS^+^, and Fe^3+^ absorbance of extract. The difference in absorbance value is caused by the presence of antioxidants in each extract. IC_50_ was calculated from a calibration curve obtained by plotting percentage inhibition versus extract concentration. A lower IC_50_ value would reflect greater antioxidant activity. The IC_50_ value of *L. bicolor* honey extract for scavenging DPPH^•^ and ABTS^+^ radicals and the reduction in Fe^3+^ was 0.2 ± 0.05 mg/g of honey, 0.5 ± 0.04 mg/g of honey, and 0.5 ± 0.01 mg/g of honey, respectively, which are comparable to vitamin C (Figure 2). As reported, the IC_50_ values of other types of honey such as spruce honey, fir honey, chestnut honey, and multifloral honey for scavenging DPPH^•^ radicals were 7.2–10.7 mg/g of honey [35]. And the IC_50_ value of *Astragalus membranaceus var. mongholicus* Hsiao honey for scavenging ABTS^+^ radical was 3.3 g/mL of honey [36]. The antioxidant capacity of these honeys was lower than that of *L. bicolor* honey. Our results suggest that *L. bicolor* honey extract could be potentially advantageous as a bioactive supplement for exerting antioxidative functions.

### 3.4. Effect of L. bicolor Honey Extract on RAW 264.7 Cells Viability

**Figure 3:**
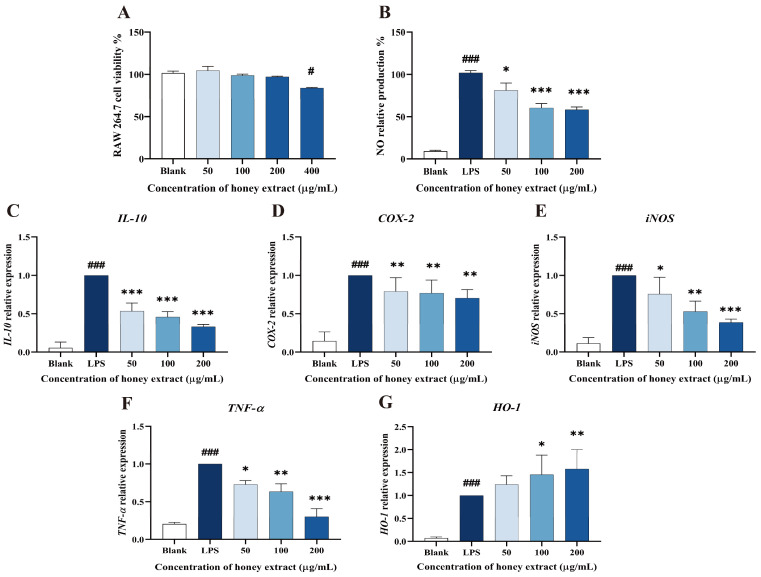
Effect of *L. bicolor* honey extract on cell viability, NO production in RAW 264.7 cells, and the mRNA expression of key inflammation- and oxidation-related cytokine genes in LPS-stimulated RAW 264.7 cells. (**A**) Effects of *L. bicolor* honey extract on the viability of RAW 264.7 cells. RAW 264.7 cells were treated with the indicated concentrations of *L. bicolor* honey extract for 24 h. *#* *p* ˂ 0.05 compared to the blank group. (**B**) Effects of *L. bicolor* honey extract on LPS-induced NO production in RAW 264.7 cells. Cells were pretreated with/without the indicated concentrations of *L. bicolor* honey extract for 1 h and then stimulated with LPS (1 µg/mL) for 24 h. LPS group referring to cells were treated with LPS in the absence of *L. bicolor* honey extract. The blank group referring to cells was treated with neither LPS nor *L. bicolor* honey extract. The relative transcript levels of (**C**) *IL-10*, (**D**) *COX-2*, (**E**) *iNOS*, (**F**) *TNF-α*, and (**G**) *HO-1* were quantified using qRT-PCR. Cells were pretreated with *L. bicolor* honey extract at the indicated concentrations for 1 h and then stimulated with LPS (1 µg/mL) for 6 h. ### *p* ˂ 0.001, compared to the blank group, which was not treated with *L. bicolor* honey extract or LPS; * *p* ˂ 0.05, **p ˂ 0.01, and ***p ˂ 0.001, compared to LPS group.

As shown in Figure 3, 50–200 µg/mL of *L. bicolor* honey extract in treatment groups did not show significantly decreased cell viability when compared to the blank control (*p* > 0.05, Figure 3A). A dose of 400 µg/mL of *L. bicolor* honey extract apparently (*p* < 0.05) decreased cell viability. Therefore, the 50, 100, and 200 µg/mL of treated *L. bicolor* honey extracts were kept for the following experiments.

### 3.5. Influence of NO Production Treating with L. bicolor Honey Extract on RAW 264.7 Cells Inducing by LPS

Macrophages may secrete excessive amounts of NO, activating transmembrane molecular signals that can harm the surrounding tissues and organs during the inflammation process [37]. LPS stimulation can promote the process of NO production and inflammation in RAW 264.7 cells. Therefore, NO production is usually taken as an indicator to evaluate the inflammatory degrees of LPS-induced RAW 264.7 cells. A concentration-dependent inhibition of LPS-treated RAW 264.7 cells on NO production was observed (Figure 3B). Specifically, pretreatment with *L. bicolor* honey extract of 50, 100, and 200 µg/mL treatment groups caused significantly decreased NO production (*p* < 0.05) when compared with the LPS group. This suggests that *L. bicolor* honey extract has the potential to inhibit NO release and mitigate the inflammation response induced by LPS.

### 3.6. L. bicolor Honey Extract Affects the Oxidation- and Inflammation-Response-Related Genes’ Expression

*L. bicolor* honey extract pretreatment was observed to have a dose-dependent effect on the cytokines expression in RAW 264.7 cells induced by LPS (Figure 3), significantly reducing the expression levels of *COX-2*, *IL-10*, *TNF-α*, and *iNOS* in the cells treated by LPS (*p* < 0.05, Figure 3C–F). Conversely, pretreatment with 100 µg/mL of *L. bicolor* honey extract led to the significant activated *HO-1* expression (*p* < 0.05) in comparison with the treatment only by LPS (Figure 3G). The reduction in *IL-10*, *COX-2*, *iNOS*, and *TNF-α* expression and increase in *HO-1* expression indicate that cells treated with *L. bicolor* honey extract show improved resistance to LPS-induced oxidative stress, highlighting its potential as both an anti-inflammatory and antioxidative agent.

### 3.7. Effect of L. bicolor Honey Extract on Cell Metabolism

**Figure 4:**
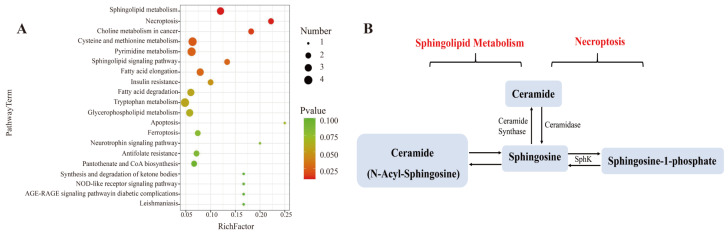
Pretreatment with *L. bicolor* honey extract alters sphingolipid and necroptosis metabolism in LPS-treated RAW 264.7 cells. (**A**) Enrichment analysis of the metabolic pathway using MetaboAnalyst 4.0 for *L. bicolor* honey treatment group vs. LPS treatment group. (**B**) Differentially regulated sphingolipid metabolism and necroptosis pathway metabolites. The metabolite nodes in red indicate the compound up-regulated in LPS-treated group compared to the blank group, but it was down-regulated in honey-treated group compared to the LPS group.

To better learn the metabolomic differences among blank, LPS, and three *L. bicolor* honey extract treatment groups, we analyzed the cellular metabolites using UHPLC-Q-TOF-MS/MS. Principal Component Analysis (PCA) showed that the blank, LPS, and *L. bicolor* honey extract treatment groups were well separated with extensive overlap between the concentration of 100 and 200 µg/mL treated groups (Figure S1A). We then compared the metabolomic profiles between blank and LPS groups and between the 100 µg/mL *L. bicolor* honey extract treatment and LPS groups, with a filtration threshold of *p* ˂ 0.05, |log2FC| ˃ 2 (Figure S1B). Different metabolites were analyzed using the KEGG pathway enrichment analysis. The analysis revealed that sphingolipid metabolism and the necroptosis pathway were repressed by *L. bicolor* honey extract on RAW 264.7 cells treated with LPS (Figure 4). Specifically, ceramide, sphingosine, and sphingosine-1-phosphate (S1P) were promoted in the RAW 264.7 cells treated with LPS, and this increase was greatly attenuated with pretreatment with *L. bicolor* honey extract (Table 2).

Ceramide is decarboxylated by ceramide synthetase after which it is metabolized into sphingosine and other inflammatory cytokines, like *TNF-α* or *IL-1β* [38], and they could activate sphingosine kinases (SphKs) including SphK-1 and SphK-2, which could produce S1P from sphingosine [39]. S1P, as a signaling molecule, works in regulating many physiological and pathological activities, including apoptosis, cell proliferation, and regulating inflammation and cytokines involved in autoimmunity [40]. S1P has been demonstrated to be involved in suppressing proinflammatory cytokine production and inducing anti-inflammatory expression in macrophages [41]. In this study, RAW 264.7 cells showed enhanced production of sphingosine following LPS stimulation, which induced the inflammatory response. However, pretreatment with *L. bicolor* honey extract inhibited the inflammatory response and down-regulated sphingosine production.

*L. bicolor* honey extract inhibited the conversion of ceramide to sphingosine and the release of other inflammatory cytokines, meanwhile, it promoted the production of S1P originating from sphingosine. S1P is an intracellular second messenger that can promote cell survival and proliferation and inhibit ceramide-induced apoptosis. Dynamic equilibrium between intracellular S1P and ceramide is very important for cell apoptosis [42]. Our results confirm that *L. bicolor* honey extract can bring the imbalance back to a normal level. Further phenotypic studies of mouse models have reported that LPS not only induced inflammation in mouse macrophages but also induced acute lung injury in mice [43]. Acute lung injury as an inflammatory response syndrome causes dysfunction of immune cells and an imbalance of immune factor secretion. Honey can inhibit lung inflammation and oxidative stress by reducing the level of inflammatory factors and has a protective effect on lung injury in mice [44]. The *L. bicolor* honey has been demonstrated to possess anti-inflammatory and antioxidant capabilities in a RAW 264.7 cell model induced by LPS, thereby exhibiting potential antioxidant activities in vivo.

## 4. Conclusions

This is the first report about the antioxidant and anti-inflammatory effects of *L. bicolor* honey to RAW 264.7 cells induced by LPS. Based on UHPLC-QQQ-MS analysis, *L. bicolor* honey contained 12 phenolic substances for its biological activity research. *L. bicolor* honey extract exerted excellent free radical (DPPH^•^ and ABTS^+^) scavenging and the ability to reduce Fe^3+^ to resist oxidative damage. It was also found that *L. bicolor* honey extract pretreatment could reduce NO production; suppress the expression of *IL-10*, *COX-2*, *iNOS*, and *TNF-α*; as well as upregulate the *HO-1* expression. In addition, pretreatment with *L. bicolor* honey extract reduced the activation of the sphingolipid metabolism and necroptosis pathways in RAW 264.7 cells. These results provide some scientific basis for further applications of *L. bicolor* honey as a natural antioxidant and anti-inflammatory agent to benefit human health.

## Figures and Tables

**Table 1:** The contents of major polyphenols in *L. bicolor* honey were detected using UHPLC-QQQ-MS/MS approach.

No.	Compound	Formula	RT/min	Regression Equation	R^2^	mg/kg (Mean ± SD)
1	Chlorogenic acid	C_16_H_18_O_9_	1.4	y = 21,979x + 3130	0.9979	16.28 ± 5.68
2	Ferulic acid	C_10_H_10_O_4_	3.3	y = 6875x − 4	0.9999	1.42 ± 0.64
3	Vitexin	C_21_H_20_O_10_	4.3	y = 21,424x − 121	0.9998	9.59 ± 2.79
4	Rutin	C_27_H_30_O_16_	3.6	y = 17,807x − 508	0.9963	7.22 ± 2.90
5	Gallic acid	C_7_H_6_O_5_	1.4	y = 2708x − 20	0.9924	5.04 ± 1.93
6	Myricitrin	C_15_H_10_O_8_	3.4	y = 41x − 2	0.9949	5.43 ± 3.52
7	Morin	C_15_H_10_O_7_	3.6	y = 440,451x + 1211	0.9963	4.39 ± 3.08
8	Kaempferol-3-O-sophoroside	C_33_H_40_O_21_	4.0	y = 3201x − 5	0.9996	7.21 ± 1.94
9	Glycitein	C_16_H_12_O_5_	4.8	y = 6452x + 272	0.9968	17.27 ± 3.37
10	Wogonin	C_16_H_12_O_5_	4.8	y = 50,170x + 2956	0.9940	7.07 ± 4.72
11	Butin	C_15_H_12_O_5_	4.0	y = 65,503x − 253	0.9972	2.77 ± 1.09
12	Liquiritigenin	C_15_H_12_O_4_	4.8	y = 126,010x − 67	0.9999	3.60 ± 1.33

**Table 2:** Changes in sphingolipid content on RAW 264.7 cells induced by LPS resulting from incubation of *L. bicolor* honey extract.

Pathway	Metabolites	LPS Group vs. Blank Group ^a^			Honey Group ^b^ vs. LPS Group ^c^		
		*p*-Value	Log2 (Fold Change)	Trend	*p*-Value	Log2 (Fold Change)	Trend
Sphingolipid Metabolism/Necroptosis	Ceramide	0.0087	13.36	up	0.0007	−2.38	down
	Sphingosine	0.0040	2.26	up	0.0380	−2.22	down
	Sphingosine-1-phosphate (S1P)	0.0005	13.42	up	0.0157	−4.77	down

^a^ Blank group was not treated with *L. bicolor* honey extract or LPS; ^b^ honey group was pretreated with 100 µg/mL of *L. bicolor* honey extract, then treated with 1 µg/mL of LPS; ^c^ LPS group was treated with 1 µg/mL of LPS but not pretreated with *L. bicolor* honey extract.

## Data Availability

Data is contained within the article and Supplementary Materials.

## Supplementary Materials

The following supporting information can be downloaded at https://www.mdpi.com/article/10.3390/antiox12101809/s1, Figure S1: Comparison of metabolite profiles of LPS-treated RAW 264.7 cells with or without pre-treatment with honey extract. (A) PCA analysis for cell metabolites from different treatment groups. (B) Volcano plot analysis for identifying the differences in metabolic profiles between different groups. Blue and red points referred to the down-regulated and up-regulated metabolites with significant differences in relative abundance between the indicated paired groups, respectively. The Blank group was not treated with *L. bicolor* honey extract or LPS; the LPS group was treated with 1 µg/mL LPS but not pretreated with *L. bicolor* honey extract; Honey group was pretreated with 100 µg/mL *L. bicolor* of honey extract and then stimulated by 1 µg/mL LPS; Table S1: Quantitative analysis of targeted polyphenol compounds by UHPLC-QQQ-MS; Table S2: Gene-specific primers for targeted cytokines; Table S3: Polyphenol and flavonoid content and antioxidant activity of *L. bicolor* honey extract; Table S4: Qualitative analysis of polyphenol compounds by UHPLC/Q-TOF-MS.
