# Supplementary material for: Antioxidant Polyphenols from Lespedeza bicolor Turcz. Honey: Anti-Inflammatory Effects on Lipopolysaccharide-Treated RAW 264.7 Macrophages
Source: Antioxidants (Basel). 2023 Sep 28;12(10):1809. doi: 10.3390/antiox12101809 (PMC10604429; doi:10.3390/antiox12101809)
Supplement: Supplementary file 1 [file antioxidants-12-01809-s001.zip › antioxidants-2565520-supplementary.pdf]

Supplementary

Table S1. Quantitative analysis of targeted polyphenol compounds by UHPLC-QQQ-MS

No.	Compound	Retention time (min)	Precursor Ion (m/z)	Product Ion (m/z)	Fragmentor (V)	Collision Energy (V)	Linear range	R^2	LOD[a] (mg/kg)	LOQ[b] (mg/kg)
1	Chlorogenic acid	1.4	353.0	190.9*<br>176.8	380	20<br>40	0.02-20	0.9979	0.005	0.020
2	Ferulic acid	3.3	193.1	177.9*<br>134.2	380	10<br>20	0.09-5	0.9999	0.025	0.090
3	Vitexin	4.3	431.0	311.2*<br>283.1	380	20<br>30	0.20-10	0.9998	0.050	0.200
4	Rutin	3.6	609.0	301.0*<br>271.0	380	40<br>58	0.04-10	0.9963	0.010	0.040
5	Gallic acid	1.4	169.0	125.0*<br>79.0	380	15<br>19	0.09-10	0.9924	0.025	0.090
6	Myricitrin	3.4	317.0	178.9*<br>151.0	380	20<br>25	0.20-10	0.9949	0.050	0.200

7	Morin	3.6	301.0	151.0*<br>125.0	380	16<br>16	0.04-5	0.9963	0.010	0.040
8	Kaempferol-3-O-sophoroside	4.0	611.0	286.6*	380	40	0.25-50	0.9996	0.025	0.090
9	Glycitein	4.8	283.0	139.8*<br>162.8	380	22<br>26	0.09-20	0.9968	0.050	0.200
10	Wogonin	4.8	283.0	268.1*<br>239.8	380	10<br>26	0.04-10	0.9940	0.010	0.040
11	Butin	4.0	273.1	137.0*<br>163.0	380	30<br>20	0.05-2.5	0.9972	0.010	0.040
12	Liquiritigenin	4.8	257.1	137.0*<br>147.0	380	30<br>20	0.05-2.5	0.9999	0.025	0.090

Note: * Product ion for quantitation. [a] mean limit of detection (LOD); [b] mean limit of quantification (LOQ). LOD and LOQ of the developed method were determined as the concentration of signal to noise ratios 3 and 10, respectively.

Table S2. Gene-specific primers for targeted cytokines

Gene	Primers	Size (bp)	No.
GAPDH	5'-GAGAAACCTGCCAAGTATGATGAC-3'	212	NM_008084.2
	5'-TAGCCGTATTCATTGTCATACCAG-3'		
IL-6	5'-CTCTGCAAGAGACTTCCATCC-3'	210	NM_031168.1
	5'-GAATTGCCATTGCACAACTC-3'		
IL-10	5'-CTATGCTGCCTGCTCTTACTG-3'	221	NM_010548.2
	5'-CAACCCAAGTAACCCTTAAAGTC-3'		
COX-2	5'-GAAATATCAGGTCATTGGTGGAG-3'	237	NM_011198.3
	5'-GTTTGGAATAGTTGCTCATCAC-3'		
iNOS	5'-TTTCCAGAAGCAGAATGTGACC-3'	294	NM_010927.3
	5'-AACACCACTTTCACCAAGACTC-3'		
TNF-α	5'-CTCCAGTGGCTGAACCGC-3'	187	NM_000594.3
	5'-GGTAGGAGACGGCGATGC-3'		

Table S3. Polyphenol and flavonoid content and antioxidant activity of *L. bicolor* honey extract

Index	Content/IC_{50}	Unit
Total polyphenols	147.7 ± 3.3	μg GAE/g honey
Total flavonoids	8.5 ± 0.8	μg QE/g honey
$DPPH^{\bullet}$	0.2 ± 0.05	mg/g honey
$ABTS^{+}$	0.5 ± 0.04	mg/g honey
FRAP	0.5 ± 0.01	mg/g honey

Table S4. Qualitative analysis of polyphenol compounds by UHPLC/Q-TOF-MS

No.	Compound	Formula	Retention time (min)	m/z (calculated)	m/z (observed)	Mass error (ppm)
1	Chlorogenic acid	$C_{16}H_{18}O_9$	1.4	353.0878	353.0895	4.81
2	Ferulic acid	$C_{10}H_{10}O_4$	3.3	193.0506	193.0505	-0.52
3	Vitexin	$C_{21}H_{20}O_{10}$	4.3	431.0984	431.0985	0.23
4	Rutin	$C_{27}H_{30}O_{16}$	3.6	609.1461	609.1490	4.76
5	Gallic acid	$C_7H_6O_5$	1.4	169.0142	169.0143	0.59
6	Myricitrin	$C_{15}H_{10}O_8$	3.4	317.0303	317.0309	1.89
7	Morin	$C_{15}H_{10}O_7$	3.6	301.0354	301.0361	2.33
8	Kaempferol-3-O-sophoroside	$C_{21}H_{30}O_{16}$	4.0	609.1461	609.1458	-0.49
9	Glycitein	$C_{16}H_{12}O_5$	4.8	283.0612	283.0604	-2.83
10	Wogonin	$C_{16}H_{12}O_5$	4.8	283.0612	283.0604	-2.83
11	Butin	$C_{15}H_{12}O_5$	4.0	271.0612	271.0618	2.21
12	Liquiritigenin	$C_{15}H_{12}O_4$	4.8	255.0663	255.0668	1.96

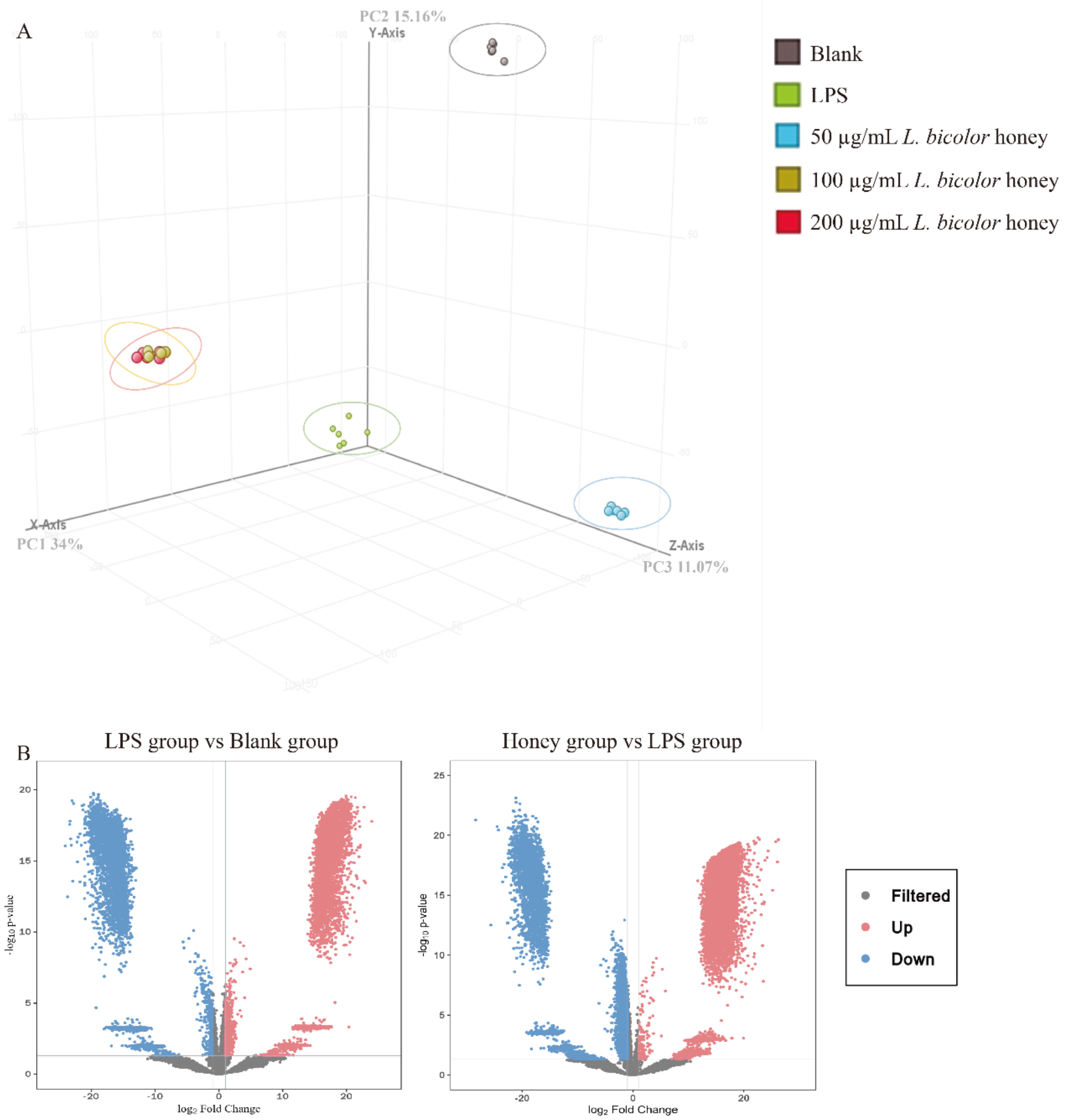

Figure S1. Comparison of metabolite profiles of LPS-treated RAW 264.7 cells with or without pre-treatment with honey extract. (A) PCA analysis for cell metabolites from different treatment groups. **(B)** Volcano plot analysis for identifying the differences in metabolic profiles between different groups. Blue and red points referred to the down-regulated and up-regulated metabolites with significant differences in relative abundance between the indicated paired groups, respectively. The Blank group was not treated with *L. bicolor* honey extract or LPS; the LPS group was treated with 1 µg/mL LPS but not pretreated with *L. bicolor* honey extract; Honey group was pretreated with 100 µg/mL *L. bicolor* of honey extract and then stimulated by 1 µg/mL LPS.
